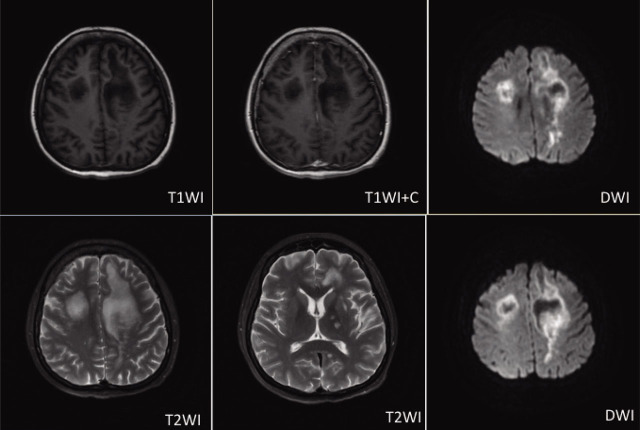# Progressive Multifocal Leukoencephalopathy in a Patient with Systemic Lupus Erythematosus

**DOI:** 10.2478/rir-2022-0024

**Published:** 2022-10-20

**Authors:** Lin Qiao, Ziang Pan, Jin Huang, Jiuliang Zhao, Chanyuan Wu, Li Wang, Mengtao Li, Yan Zhao

**Affiliations:** 1 Department of Rheumatology and Clinical Immunology, Chinese Academy of Medical Sciences& Peking Union Medical College; National Clinical Research Center for Dermatologic and Immunologic Disease (NCRC-DID), Ministry of Science & Technology; State Key Laboratory of Complex Severe and Rare Diseases, Peking Union Medical College Hospital (PUMCH); Key Laboratory of Rheumatology and Clinical Immunology, Ministry of Education, Beijing, 100730, China; 2 Department of Neurology, Chinese Academy of Medical Sciences& Peking Union Medical College, Peking Union Medical College Hospital (PUMCH), Beijing 100730, China; 3 Department of Internal medicine, Chinese Academy of Medical Sciences& Peking Union Medical College, Peking Union Medical College Hospital (PUMCH), Beijing 100730, China

A 36-year-old female was admitted because of arthritis for 5 years and aphasia for 1 month. She was diagnosed with systemic lupus erythematosus (SLE) and treated with oral steroids and cyclosporine. One year before admission, she developed fever, hypocomplementaemia, and a high-titer anti-double-stranded DNA antibody (anti-dsDNA), and received belimumab and IL-2 for 6 months. However, her linguistic ability and physical activity deteriorated rapidly. On admission, the laboratory tests showed lymphocytopenia (0.11–0.27 × 10^9^/L) and elevated HbA1c (9.0%). Brain magnetic resonance imaging (MRI) showed extensive cerebral lesions. Multiple patchy-like lesions were seen in the left basal ganglia, bilateral periventricular white matter, and centrum semiovale, and parts of subcortical U-shaped fibers were involved. The lesions were hypointense on T1WI, hyperintense on T2WI with rounded hyperintensity on diffusion weighted imaging (DWI), and without noticeable enhancement on T1 contrast. The cell count and biochemistry of the cerebrospinal fluid were normal. A specific oligoclonal band was negative. JC polyomavirus (JCV)-DNA was positive in her cerebrospinal fluid and urine. She was diagnosed with JCV-induced progressive multifocal leukoencephalopathy (PML). The steroids were tapered and immunosuppressants were temporarily stopped. And we gave a course of immunoglobulin for immune reconstitution. For personal reasons, she was discharged against medical advice.

PML has primarily been described in severely immunosuppressed patients.^[1]^ The use of immunosuppressants in SLE patients may contribute to an increased risk of PML. It is a great challenge for physicians to differentiate PML from neuropsychiatric SLE. Typical hyperintense lesions in T2 and FLAIR sequences in the brain MRI strongly support the diagnosis of PML.^[2]^ The detection of JCV in cerebrospinal fluid by PCR is a diagnostic confirmation of PML, though the definite diagnosis is only made by brain biopsy. Therapeutic management is very complex, and two strategies should be used concomitantly. First, trying to eliminate the virus; although there is no approved therapeutic method specific for patients that do not have HIV.^[3]^ Second, immunosuppressants that cause lymphopenia should be removed, simultaneously avoiding aggravating SLE with their withdrawal.

**Figure:**